# Genome wide identification and functional assignments of C_2_H_2_ Zinc-finger family transcription factors in Dichanthelium oligosanthes

**DOI:** 10.6026/97320630015689

**Published:** 2019-10-16

**Authors:** Manisha Mahapatra, Bijayalaxmi Mahanty, Raj Kumar Joshi

**Affiliations:** 1 Department of Biotechnology, Rama Devi Women's University, Vidya Vihar, Bhubaneswar-751022, Odisha, INDIA

**Keywords:** Zinc fingers, C_2_H_2_-ZFPs, transcription factors, phylogenetic analysis, Dichanthelium oligosanthes

## Abstract

Transcription factors (TFs) are biological regulators of gene function in response to various internal and external stimuli. C_2_H_2_ zinc finger proteins (C_2_H_2_-ZFPs) are a large family of TFs that play crucial roles in plant growth and development, hormone signalling and response to biotic and abiotic stresses. While C_2_H_2_-ZFPs have been well characterized in many model and crop plants, they are yet to be ascertained in the evolutionarily important C_3_ plant Dichanthelium oligosanthes (Heller's rosette grass). In the present study, we report 32 C_2_H_2_-ZF genes (DoZFs) belonging to three different classes-Q type, C-type and Z-type based on structural elucidation and phylogenetic analysis. Sequence comparisons revealed paralogs within the DoZFs and orthologs among with rice ZF genes. Motif assignment showed the presence of the distinctive C_2_H_2_-ZF conserved domain "QALGGH" in these proteins. Cis-element analysis indicated that majority of the predicted C_2_H_2_-ZFPs are associated with hormone signalling and abiotic stress responses. Further, their role in nucleic acid binding and transcriptional regulation was also observed using predicted functional assignment. Thus, we report an overview of the C_2_H_2_-ZF gene family in D. oligosanthes that could serve as the basis for future experimental studies on isolation and functional implication of these genes in different biological mechanism of C_3_ plants.

## Background

Transcription factors (TFs) are regulatory proteins which play critical role in altering the expression of genes associated with multiple cellular pathways related to growth, development and stress responses [1]. Among the various TFs, the Zinc-finger proteins (ZFPs) are the largest group of transcription regulators in plants [2]. ZFPs constitute a two stranded antiparallel beta sheet and a helix stabilized by zinc finger domains consisting of zinc ion surrounded by cysteine and histidine residues. Since the discovery of the first ZFP from Petunia, several zinc-finger TFs have been identified from myraids of plants and their involvement in different biological processes including growth, development, reproduction, photosynthesis and stress responses have been reported [2].

Among all the ZFP types, C_2_H_2_-ZFPs are the most widely distributed transcription factors in eukaryotes. These are characterized by the presence of a conserved motif X_2_-Cys-X_2_-_4_-Cys-X_12_-His-X_3_-_5_-His, where X represents the amino acids that act as the spacer between the cysteine and the histidine residues [3]. Experimental analyses have shown that C_2_H_2_-ZFPs are represented by 3% of all genes in mammals, 2.3% of all the genes in Drosophila and 0.8% of all genes in yeast [4].Compared to other eukaryotes, the plant C_2_H_2_-ZFPs are characterized by the presence of highly conserved QALGGH motif in the zincfinger helices and have long spacers with variable length and sequence between the zinc finger domains [2,4]. Extensive identification and characterization of C_2_H_2_-ZFPs have been reported in plants including 179 from Arabidopsis [5], 189 in rice [6], 124 in foxtail millet [7], 109 in Populus trichocarpa [8] and 122 in durum wheat [9]. Accumulating evidences indicate that C_2_-_4_-ZFPs are critically associated with transcriptional regulation, RNA metabolism and protein-protein interactions [10,11].A wide number of plant C_2_-_4_-ZFPs have been functionally implicated in multiple physiological processes including floral organogenesis [12], growth initiation [13], biogenesis of non-coding RNAs [14], abiotic stress responses [15,16], pathogen defence [17].

Dichanthelium oligosanthes, also known as the Heller's rosette grass is a frost tolerant perennial wild penicoid grass species which utilizes the C_3_ pathway for carbon fixation and lacks Kranz anatomy [18]. Therefore, it can be used as a model species to understand the evolutionary developmental pattern of C_4_ photosynthesis when compared with important C_4_ relatives, including rice, wheat, and maize. The draft genome of D. oligosanthes has been recently sequenced and a small suite of transcription factors associated with C_4_ photosynthesis have been identified [19]. While, extensive studies of C_2_-_4_-ZFPs and their association with biological and physiological mechanisms have been conducted in many plant species, no report is available from D. oligosanthes so far. Therefore, it is important to perform a genome-wide identification and characterization of C_2_-_4_-ZF family of transcription factors to illuminate their molecular role in D. oligosanthes. In the present study, we identified 32 C_2_H_2_-ZF genes from D. oligosanthes utilizingvaried bioinformatics tools. The structural organization of the identified genes including exon-intron arrangements, 5'/3' untranslated regions (UTRs), conserved protein motifs and promoter cis-elements were determined. Further, the identified proteins were analyzed for their phylogenetic relationship and orthology/ paralogy within D. oligosanthes as well as with other model plant species. Additionally, the functional characteristics of the identified C_2_-_4_-ZFPs were predicted using gene ontology (GO) analyses. These results will form the basis for future gene functional studies of C_2_-_4_-ZFPs in towards understanding physiological responses in D. oligosanthes.

## Methodology

### Identification and characteristics of C_2_H_2_-ZF gene family

The draft genome sequence of D. oligosanthes (ASM163321v2) was downloaded from NCBI database (http:// www.ncbi.nlm.nih.gov/). The hidden Markov model (HMM) profile of C_2_H_2_-ZF (PF00096) was downloaded from the Protein family (Pfam) database (http://pfam.xfam.org/) and subsequently used as a query in the HMMER database (https://www.ebi.ac.uk/Tools/hmmer) to search for C_2_H_2_-ZF proteins in D. oligosanthes. The retrieved candidate protein sequences were further analyzed with the SMART (http://smart.embl-heidelberg.de/) database to confirm the presence of C_2_H_2_-ZF domain in the sequences. Specific properties of the deduced popypeptides including molecular weight, isoelectric points and hydropathy were calculated using the ExPaSy site (http://web.expasy.org/protparam/).

### Sequence alignment and phylogenetic analysis

C_2_H_2_-ZF gene and protein sequences from model plant Arabidopsis and rice were obtained from The Arabidopsis Information Resource (TAIR, http://www.arabidopsis.org/index.jsp) and Rice Genome Browser (http://www.tigr.org/tigr-scripts/osa1web/gbrowse/ rice) respectively. Multiple sequence alignment of the full length C_2_H_2_-ZF protein sequences from D. oligosanthes, A. thaliana and O. sativa was performed using Clustal Omega (https://www.ebi.ac.uk/Tools/msa/clustalo/) with default parameter and manually adjusted using BioEdit 7.1 software [20]. Phylogenetic analyses of the protein sequences were performed using Molecular Evolutionary Genetic Analysis (MEGA v 10.1) package [21]. A neighbourjoining (NJ) method with 1000 bootstrapping was performed to develop an unrooted phylogenetic tree.

### Structural organization and identification of conserved motifs

The individual cDNA sequences of the C_2_H_2_-ZF genes and their corresponding genomic sequences were compared using the Gene Structures Display Server (GSDS 2.0; http://gsds.cbi.pku.edu.cn/index.php) to generate the intron/exon organization. Motif structures of the predicted protein were analyzed using Multiple Expectation Maximization for motif Elicitation (MEME) tool [22] using the set parameters as follows: occurrence of motif repeats: any number, max number of motifs to be predicted: 20, and Min/Max motif width: 10/100.

### Promoter cis-element analysis and identification of paralogs and orthologs

Promoter sequences about 2Kb upstream of the translation start site for all the C_2_H_2_-ZF genes were obtained from the NCBI database.

The cis-acting regulatory elements were located and predicted from the putative C_2_H_2_-ZF promoter regions by using Plant-CARE [23]. All the cDNA sequences of the C_2_H_2_-ZFgenes were compared amongst themselves (all-against-all) by performing BLASTn to identify the paralogous ZFs in D. oligosanthes. After each round of BLASTn, sequences showing ≥ 40% sequence similarity with at least 300bp sequence alignment were considered to be paralogous [24]. To predict the orthologs in rice, each of the rice C_2_H_2_-ZF sequences was used as a query to search against all DoZF sequences by using BLASTn. The BLASTn results showing the best hits with at least 300 bp region of alignment with a DoZF was considered to be an ortholog [24].

### Sub-cellular localization and gene ontology (GO) analysis

The subcellular localization of C_2_H_2_-ZF proteins was predicted using the mGOASVM (Plant V2) server [25]. The functional grouping of C_2_H_2_-ZF sequences from D. oligosanthes and the annotation data were computed using the Blast2GO v3.0 [26] and cross verified using the DeepGO protein function prediction tool with the protein GO classes [27]. Blast2GO annotation associates genes or transcripts with GO terms classified into three categories: biological processes, molecular functions and cellular components.

## Results and Discussion

The HMM profile of the C_2_H_2_-ZF domain (PF00096) was used as a query to search for C_2_H_2_-ZF genes of D. oligosanthes within the protein databases using HMMER software. A total of 57 C2H2-ZF genes were obtained. A recent study using similar approach identified 14 Squamosa promoter-binding protein-like (SPL) TFs in D. oligosanthes [28]. The candidate sequences thus obtained were analysed using the Simple Modular Architecture Research Tool (SMART; SM000355) and the Conserved Domain Database (CDD) to validate the presence of C_2_H_2_ ZFs. Finally, 32 C_2_H_2_-ZF genes were identified and names as DoZFP1 to DoZFP32 (C_2_H_2_ ZFPs of D. oligosanthes). This number is quite less than those found in Arabidopsis, rice, foxtail millet and Populus [5-8]. Analysis of the peptide properties showed that DoZFPs had molecular masses ranging from 21133.18 Da (DoZF2) to 166234.58 Da (DoZF13). Likewise, the length of the amino acids in the encoded proteins of DoZFPs greatly varied between 196aa (DoZF3) to 15103aa (DoZF13). Also, the pI values of the predicted proteins ranged between 5.53 (DoZF2) to 10.11 (DoZF21). Subcellular localization using mGOASVM revealed that all except one C_2_H_2_ZFPs were predicted as nuclear proteins while only DoZFP4 was located in the endoplasmic reticulum. Additionally, the hydropathy plot obtained from Expasy protscale revealed that majority of the identified DoZFPs were basic in nature (data not shown). Also, 26 DoZFs were basic while the remaining 6 predicted proteins were found acidic in nature. The details of the properties of the DoZFP nucleic acid and protein sequences are represented in Table 1.

**Figure 1:**
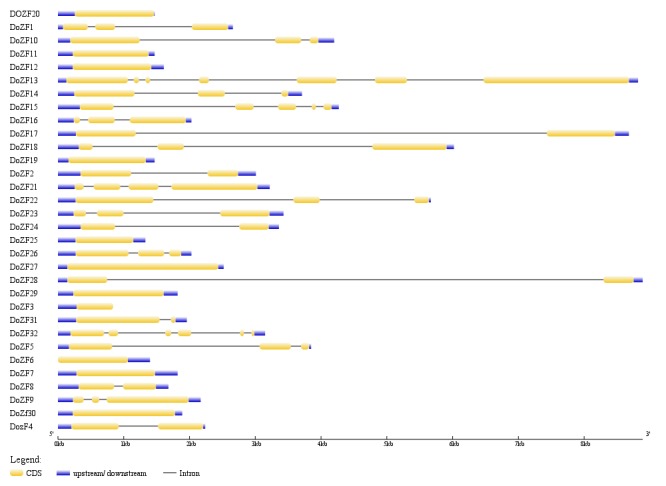
Gene structure analysis of D. oligosanthes C_2_H_2_ ZF genes. Exon/intron structures were obtained from the Gene Structure Display Server. Exons, introns and the UTR regions of each gene are represented by yellow boxes, black lines and blue boxes, respectively.

Diversity of the gene structure, cis-regulatory elements and conservation of protein motifs is possible instrument for the evolution of gene families in plants [29]. The intron/exon organization of the DoZFPs was determined by comparing the coding sequence with their corresponding genomic DNA sequences using GSDS software. The number of exons varied from 1 (DoZF3, DoZF6, DoZF7, DoZF11, DoZF12, DoZF19, DoZF20, DoZF25, DoZF29, DoZF30) to 7 (DoZF13) with 13 DoZFPs composed of three or more exons (Figure 1). In contrast, 14 DoZFPs had two or more than two introns while 10 DoZFPs had no introns. Similar organization of introns/exon organization has been reported for C_2_H_2_-ZFPs in Populus and rice [6,8]. Cis regulatory elements are key factors in controlling the transcriptional regulation of genes [30]. Therefore, the interaction between key transcription factors and specific cis-element is crucial in plants' response to phyto hormones as well as biotic and abiotic stresses [31]. Promoter sequence 2000 bp upstream of the translation initiation site in the 32 DoZFP genes were examined for the presence of cis-element using the PlantCARE database. Results revealed that 1 to 11 TATA box element and 1 to 8 CAAT box elements were found in the promoter regions of 32 DoZF genes. In addition, DoZFP gene promoters contains multiple cis regulatory elements responsive to phyto hormone and stress signalling, including ABRE (Abscisic acid responsive element), TCA (Salicylic acid responsive element), MYB and MYC regions, CGTCA (Methyl jasmonate responsive element), ERE (ethylene responsive element), G-box (light responsive element), and W-box (WRKY binding draught responsive element). Similar cis-elements have been reported in the promoters of C_2_H_2_ZFPs in Arabidopsis thaliana [5] and further in-depth analysis of these regulatory regions would be needed to validate their roles in stress responsiveness of D. oligosanthes. 

**Figure 2:**
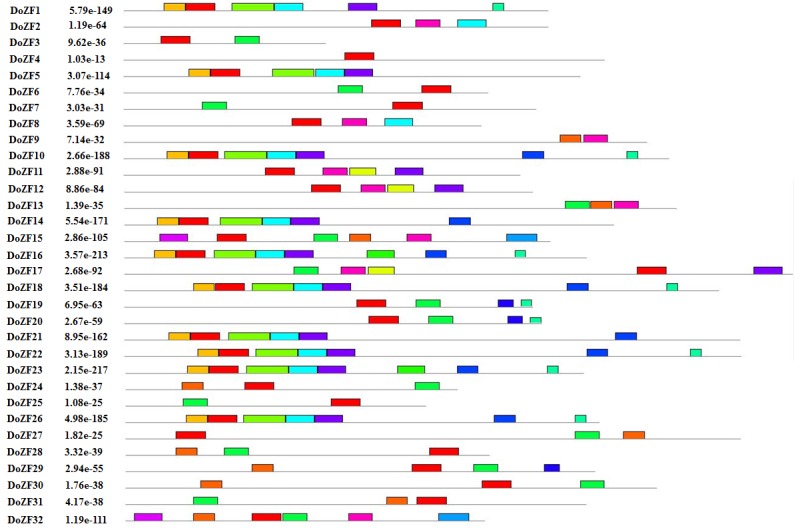
Motif composition of D. oligosanthes C_2_H_2_ ZF proteins. The conserved motifs of each gene were identified by MEME. The black lines represent the length of the protein while the color boxes represent the motif sequences represented in supplementary Table 1.

To further reveal the diversification of C_2_H_2_-ZFPs in D. oligosanthes, conserved protein motif sequences were predicted using MEME web server [22]. A total of 15 distinct structural motifs were predicted (Figure 2;Table 2). Motif 1, 2, 7 and 11 represented distinctive conserved regions of the C_2_H_2_-ZFPs. Motif 7 and 11 constituted the plant specific conserved domain "QALGGH" and were found in 11DoZFPs that were identified as Q-type. Among the Q-types, DoZF29 have a modified conserved sequence "ALGGH" and classified as M-typeC_2_H_2_-ZFP. Likewise, 15DoZFPs consisted of Motif 1 with conserved sequence "CGKGFQRDQNLQLHRRGH" and motif 2 with conserved sequence "CGKGFKRDANLRMHMRGH", the characteristic features of the Z-type C_2_H_2_-ZFPs. The remaining 6 DoZFPs (DoZFP4, DoZFP9, DoZFP13, DoZFP15, DoZFP25 and DoZFP32) did not contain any known conserved motif in the ZF region and were categorized as C-type C_2_H_2_-ZFPs. Additionally, 11 unidentified conserved motifs were also identified that were randomly placed across all the DoZFPs. Taken together, our results suggest that functionally divergent group of C_2_H_2_-ZFPs are associated in numerous plant developmental and physiological processes of D. oligosanthes.

**Figure 3:**
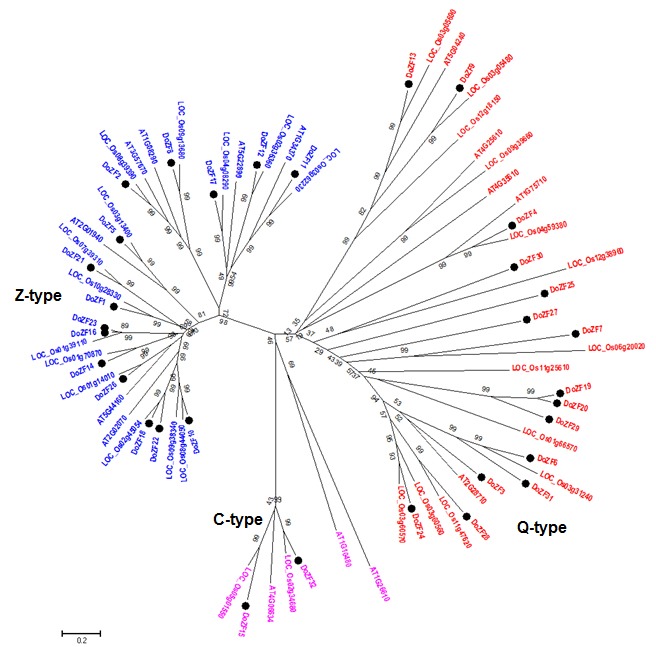
Unrooted phylogenetic tree representing the relationship among C_2_H_2_-ZFPs of D. oligosanthes, rice and Arabidopsis. The protein sequences of C_2_H_2_-ZFPs were aligned with Clustal Omega and phylogenetics tree was constructed using the neighbor-joining method in MEGA 10.0. The Bootstrap value was 1,000 replicates

To explore the evolutionary association of the identified DoZFPs,full length protein sequences of 32 DoZFPs, 15 AtZFPs and 29 OsZFPs were used to construct a neighbor-joining tree (Figure 3). The resulting tree clustered all the C_2_H_2_-ZFPs into two groups- I and II similar to previous grouping of C_2_H_2_-ZFPs reported in rice [6] and Arabidopsis [5].Group I consisted of 40 proteins including 15 Q-type DoZFPs and 2 C-type DoZFPs. Likewise, group II categorized 36 proteins including 15Z-type DoZFPs. Previous reports have shown that C-type ZFs are grouped with Z-type as well as Q-type ZFs [8]. Nevertheless, our results support the hypothesis that Q-type plant specific ZFs have evolved from C-type ZFs through conservation of the "QALGGH" sequence [6]. Further, assessment of paralogy among DoZFs and orthology of DoZFs with OsZFs revealed that 12 DoZFs were paralogous with an average of 90% similarity while 21 were orthologous (68% similarity) with OsZFs (Table 3).The genomic expansion and evolutionary divergence of a species depends on genetic duplication of functional traits [32]. Similar to C_2_H_2_-ZFPs, several TFs in different plants including NAC, WRKY and HD-Zip exhibit gene duplication as an adaptive mechanism towards dynamic environmental conditions [33,34].

Gene ontology (GO) term analyses of the predicted proteins using Blast2GO v3.0 categorized them into cellular components, molecular functions and biological processes (Table 1). Among the biological process categories, all the DoZFs represented regulation of DNA transcription (GO: 1903506) and RNA biosynthesis (GO: 2001141). Similarly, cellular component prediction showed that, 31 DoZFPs were represented by 'cell part (GO: 0044464)' while only DoZF4 was represented as 'intracellular part (GO: 0044424)'. Within the 'molecular function category', 31DoZFs were represented by GO terms 'DNA binding (GO: 0003677)' and nucleic acid binding (GO: 0003676)' suggesting their primary molecular role as interaction modules that binds to DNA, RNA and proteins [35]. In addition, DoZF4 represented transporter activity (GO: 0022891).

## Conclusion

A comprehensive genome wide analysis including phylogenetic relationships, structural prediction, conserved motif analysis and gene functions of the C_2_H_2_ZF gene family in D. oligosanthes were performed. Our analysis identified 32 C_2_H_2_ZF genes in D. oligosanthes. Phylogenetic analysis grouped the DoZFPs into three clusters similar to their orthologs in Arabidopsis and rice. Structural and motif elucidation demonstrated the presence of multiple conserved domains "QALGGH" suggesting their implication in DNA binding and transcription factor activity. Further, the cis-element analysis of the DoZFs showed their involvement in hormone signalling and stress responses. These data form the basis for functional characterization of suitable candidate genes to untangle their different roles in biological regulation.

## Figures and Tables

**Table 1:** Details protein properties of the 32 putative DoZF genes in Dichanthelium oligosanthes

Name	Accession no.	Gene Length (bp)	Protein length (aa)	pI	Mw	No. of Exons	Nature	Location	Functional annotations		
									Molecular Function	Biological Process	Cellular Component
DoZF1	A0A1E5VCE4	2660	412	8.88	45441.87	3	Basic	Nucleus	DNA binding	Regulation of DNA transcription; RNA Biosynthesis	cell part
DoZF2	A0A1E5V5R3	3010	412	5.53	44753.38	2	Basic	Nucleus	Nucleic acid binding	″	cell part
DoZF3	A0A1E5ULE0	840	196	9.19	21133.18	1	Basic	Nucleus	DNA binding	″	cell part
DoZF4	A0A1E5WJJ3	2240	467	8.86	50274.79	2	Basic	Endoplasmic	Transporter activity	″	Intracellular
								reticulum			part
DoZF5	A0A1E5UWK3	3850	443	9.42	46308.29	3	Basic	Nucleus	DNA binding	″	cell part
DoZF6	A0A1E5V146	1400	354	5.47	38591.86	1	Basic	Nucleus	DNA binding	″	cell part
DoZF7	A0A1E5UV69	1820	400	6.39	42682.01	1	Basic	Nucleus	DNA binding	″	cell part
DoZF8	A0A1E5VYM1	1680	347	6.56	37719.87	2	Basic	Nucleus	DNA binding	″	cell part
DoZF9	A0A1E5WDR2	2170	508	8.97	54709.53	3	Basic	Nucleus	DNA binding	″	cell part
DoZF10	A0A1E5UWE6	4200	529	9.23	54778.17	3	Basic	Nucleus	DNA binding	″	cell part
DoZF11	A0A1E5WHS1	1470	385	5.97	41773.84	1	Basic	Nucleus	DNA binding	″	cell part
DoZF12	A0A1E5VDK3	1610	398	6.37	41839.03	1	Acidic	Nucleus	Nucleic acid binding	″	cell part
DoZF13	A0A1E5WNK4	8820	1513	6.04	166234.58	7	Acidic	Nucleus	DNA binding	″	cell part
DoZF14	A0A1E5VGA4	3710	477	8.55	50555.38	3	Basic	Nucleus	DNA binding	″	cell part
DoZF15	A0A1E5V0A6	4270	415	7.64	46301.69	5	Basic	Nucleus	-	″	cell part
DoZF16	A0A1E5VAW9	2030	450	8.95	47981.05	3	Basic	Nucleus	DNA binding	″	cell part
DoZF17	A0A1E5W708	8680	651	9.21	70756.91	2	Basic	Nucleus	DNA binding	″	cell part
DoZF18	A0A1E5V9G2	6020	579	8.8	60159.21	3	Basic	Nucleus	DNA binding	″	cell part
DoZF19	A0A1E5UKX2	1470	398	6.71	42397.25	1	Acidic	Nucleus	DNA binding	″	cell part
DoZF20	A0A1E5V5J2	1470	407	6.55	43188.79	1	Acidic	Nucleus	DNA binding	″	cell part
DoZF21	A0A1E5VQB0	3220	766	6.31	83834.76	4	Acidic	Nucleus	Nucleic acid binding	″	cell part
DoZF22	A0A1E5W4N3	5670	601	8.77	62025.16	3	Basic	Nucleus	DNA binding	″	cell part
DoZF23	A0A1E5UV96	3430	447	8.59	47656.73	3	Basic	Nucleus	DNA binding	″	cell part
DoZF24	A0A1E5V1M3	3360	324	9.65	34570.3	2	Basic	Nucleus	DNA binding	″	cell part
DoZF25	A0A1E5W553	1330	293	9.16	32845.43	1	Basic	Nucleus	protein binding	″	cell part
DoZF26	A0A1E5VAN5	2030	462	9.15	48920.7	3	Basic	Nucleus	nucleic acid binding	″	cell part
DoZF27	A0A1E5V5X6	2520	765	6.23	78317.88	1	Acidic	Nucleus	DNA binding	″	cell part
DoZF28	A0A1E5W465	8890	355	10.11	38031.12	2	Basic	Nucleus	DNA binding	″	cell part
DoZF29	A0A1E5WH31	1820	458	6.66	47753.09	1	Basic	Nucleus	DNA binding	″	cell part
DoZF30	A0A1E5WCG2	1890	518	5.85	54428.13	1	Basic	Nucleus	DNA binding	″	cell part
DoZF31	A0A1E5WEM4	1960	449	5.88	48157.65	2	Basic	Nucleus	DNA binding	″	cell part
DoZF32	A0A1E5V0D6	3150	350	7.95	39605.33	6	Basic	Nucleus	DNA binding	″	cell part

**Table 2:** Motif sequences of C_2_H_2_-ZF genes identified in D. oligosanthes

Motif	Width (a.a.)	Best possible match	Domain
1	29	FVCEICGKGFQRDQNLQLHRRGHNLPWKL	Z-type C_2_H_2_
2	25	HSCKCGKGFKRDANLRMHMRGHGDE	Z-type C_2_H_2_
3	28	WKCDKCSKRYAVQSDWKAHSKTCGTREY	NA
4	41	APRKRVYVCPEPSCVHHDPARALGDLTGIKKHFCRKHGEKK	NA
5	29	RCDCGTLFSRRDSFITHRAFCDALAZESA	NA
6	29	PPKRKKPGTPDPDAEVIALSPRTLLATNR	NA
7	23	HECPECGKVFTSGQALGGHMRRH	Q-type C_2_H_2_
8	21	PHMSATALLQKAAQMGATTSG	NA
9	29	GCRRNREHPRFRPLKSAVCLKNHYRRSHC	NA
10	22	KCPWDGCDKAYKWSWKLNLHLK	NA
11	18	CGRSFPSYQALGGHRRSH	Q-type C_2_H_2_
12	11	MTRDFLGVGGG	NA
13	27	QQQQQQRCNYAMKTEMPPWPPMTYDHH	NA
14	19	VRLFGIDISPQVQAPSEQQ	NA
15	29	QWSGKAMYEDDSEETEEEGENNIEDGWRY	NA

**Table 3:** Paralogous and orthologous C_2_H_2_-ZF gene pairs in D. oligosanthes and Oryza sativa

PARALOGS within DoZFs	ORTHOLOGS of DoZFs in Oryza sativa
DoZF11/DoZF12	DoZF1/ LOC_Os10g28330
DoZF14/DoZF16	DoZF2/ LOC_Os08g39390
DoZF14/DoZF18	DoZF4/ LOC_Os04g59380
DoZF14/DoZF21	DoZF5/LOC_Os03g13400
DoZF14/DoZF22	DoZF6/LOC_Os03g31240
DoZF14/DoZF23	DoZF8/LOC_Os09g13680, LOC_Os08g39390
DoZF16/DoZF23	DoZF9/LOC_Os03g05480
DoZF18/DoZF22	DoZF10/ LOC_Os08g44050, LOC_Os09g38340 , LOC_Os02g45054
DoZF19/DoZF20	DoZF11/ LOC_Os03g62230
	DoZF12/ LOC_Os04g08290, LOC_Os03g62230
	DoZF14/ LOC_Os01g70870, LOC_Os01g14010, LOC_Os07g39310,
	LOC_Os09g38340, LOC_Os02g45054, LOC_Os08g44050
	DoZF15/LOC_Os05g01550
	
	DoZF16/ LOC_Os01g39110
	DoZF17/LOC_Os04g08290, LOC_Os03g62230
	DoZF18/ LOC_Os02g45054
	DoZF21/ LOC_Os07g39310
	DoZF22/LOC_Os02g45054
	DoZF23/ LOC_Os01g39110, LOC_Os01g14010, LOC_Os01g70870,
	LOC_Os09g38340, LOC_Os08g44050
	DoZF24/ LOC_Os03g60570, LOC_Os03g60560
	DoZF26/LOC_Os01g14010
	DoZF32/LOC_Os02g34680
